# Pulsed Field Ablation in Atrial Fibrillation: Initial Experience of the Efficacy and Safety in Pulmonary Vein Isolation and Beyond

**DOI:** 10.3390/jcdd11110356

**Published:** 2024-11-05

**Authors:** Julian Cheong Kiat Tay, Jannah Lee Tarranza, Shaw Yang Chia, Xuan Ming Pung, Germaine Jie Min Loo, Hooi Khee Teo, Colin Yeo, Vern Hsen Tan, Eric Tien Siang Lim, Daniel Thuan Tee Chong, Kah Leng Ho, Chi Keong Ching

**Affiliations:** 1 Department of Cardiology, National Heart Centre Singapore, Singapore 169609, Singapore; juliantay91@gmail.com (J.C.K.T.); jannah.lee.s.t@nhcs.com.sg (J.L.T.); chia.shaw.yang@nhcs.com.sg (S.Y.C.); pung.xuanming@singhealth.com.sg (X.M.P.); germaine.loo.j.m@singhealth.com.sg (G.J.M.L.); teo.hooi.hee@singhealth.com.sg (H.K.T.); eric.lim.t.s@singhealth.com.sg (E.T.S.L.); daniel.chong.t.t@singhealth.com.sg (D.T.T.C.); ho.kah.leng@singhealth.com.sg (K.L.H.); 2 Department of Cardiology, Changi, General Hospital, Singapore 529889, Singapore; colin.yeo@singhealth.com.sg (C.Y.); tan.vern.hsen@singhealth.com.sg (V.H.T.)

**Keywords:** pulsed field ablation, pulmonary vein isolation, posterior wall ablation

## Abstract

Regional differences in pulsed field ablation (PFA) adoption for pulmonary vein isolation (PVI) with additional posterior wall ablation (PWA) in Asia remains unknown. We hereby report our experience on the safety and efficacy of PFA in AF ablation. Consecutive AF patients who underwent PFA from September 2022 to January 2024 were included. The primary efficacy endpoint was freedom from atrial arrhythmia recurrence after a 90-day blanking period at 12 months. Safety endpoints included 30 days of all-cause death, cardiac tamponade, stroke, myocardial infarction, and heart failure hospitalization. One hundred and one (72.3% males, 79.2% pAF) patients underwent PFA for AF. Thirty-one (30.7%) had structural heart disease with mean LVEF of 57.4 ± 8.1% and CHA2DS2-VASc score of 1.4 ± 1.3. Twenty-nine (28.7%) underwent additional PWA (PVI + PWA) using PFA. PWA was acutely successful in all patients. Patients who underwent PWA were more likely to have persistent AF and require general anesthesia and electroanatomic mapping (all *p* < 0.05). Total PFA applications for PVI, LA dwell time, procedural time, and fluoroscopy time were similar between the PVI-only and PVI + PWA groups (all *p* > 0.05). The 1-year atrial arrhythmia recurrence rates were 10% for pAF and 21% for the persistent AF group. The primary efficacy endpoint was not significantly different between the PVI-only and PVI+PWA groups (12-month KM estimates 90.3% [95% CI, 83.3–97.3] and 82.8% [95% CI, 68.1–97.4], respectively). There were no complications related to PFA use. PFA can be safely, effectively, and efficiently adopted for AF ablation. Additional PWA, if pursued, had similar procedural metrics to the PVI-only strategy without increased complications.

## 1. Introduction

Atrial fibrillation (AF) is the most common heart rhythm disorder, with significant impacts on morbidity and mortality [1,2]. Catheter ablation, specifically pulmonary vein isolation (PVI) using thermal energy, has been shown to be an effective treatment strategy for patients with symptomatic AF [3,4]. However, residual safety concerns pertaining to the non-selective nature of tissue destruction in thermal sources, such as atrio-esophageal fistula (AEF), pulmonary vein (PV) stenosis, and phrenic nerve injury (PNI), remains [5,6].

As such, pulsed field ablation (PFA) is fast emerging as a viable alternative given its ability to preferentially target myocardial tissues with no or little damage to the surrounding structures with comparable safety [7,8,9] and efficacy [10] for PVI. It is a new technique that constitutes the evolution of years of research in the field of AF ablation to circumvent some of the limitations of thermal energy sources [11,12]. Despite this, the use of PFA beyond PVI, such as posterior wall ablation (PWA), as well as regional differences in PFA adoption in Asia, remains relatively unknown. Our aim is to describe our initial experience with the safety and efficacy of PFA for AF catheter ablation.

## 2. Materials and Methods

### 2.1. Patient Population and Workflow

This is a prospective study conducted by two Singaporean centers. Consecutive patients with symptomatic paroxysmal (pAF) or persistent AF (persAF) who either failed or did not tolerate at least one anti-arrhythmic drugs (AAD) treatment and underwent AF ablation using PFA from September 2022–January 2024 were included.

The FARAPULSE^TM^ PFA system (Boston Scientific Inc., Menlo Park, CA, USA) was first introduced in Asia in our center in September 2022. During this one-week pilot phase, 13 patients who had predominantly pAF with minimal comorbidities underwent PVI with PFA after a period of in-service training. Thereafter, the nature of cases and the pre- and intra-procedural planning for cases listed using PFA were left to the operators’ discretion.

### 2.2. Procedure

Prior to the procedure, the patients were on a continuous uninterrupted oral anticoagulation for at least 3 weeks. AADs were not required to be stopped before ablation. Left atrial (LA) imaging was performed (transesophageal echocardiography (TEE), as well as cardiac computed tomography (CT) or cardiac magnetic resonance imaging (CMR)) within 24–72 h to exclude LA thrombus. Procedures were performed under deep sedation or general anesthesia (GA), as per operators’ discretion. Bilateral femoral venous accesses were obtained with ultrasound guidance in a standard manner. A trans-septal puncture of the interatrial septum was performed using a standard 8-Fr SL0/1 long sheath with a BRK-0/1 needle to allow for the access into the LA under fluoroscopic and intracardiac echocardiography (ICE) guidance. Heparin was administered before or immediately after the trans-septal puncture and was sustained throughout the procedure to maintain activated clotting time levels of 300–350 s, with monitoring every 15 min for the duration of the procedure.

### 2.3. PFA

The creation of a baseline electroanatomic map (EAM) using any of the clinically approved mapping systems was optional, as per the operators’ discretion. Over a long guidewire, the sheath was exchanged for the 13-Fr FARADRIVE^TM^ sheath (Farapulse-Boston Scientific Inc., Menlo Park, CA, USA). The FARAWAVE^TM^ PFA catheter (Farapulse-Boston Scientific Inc., Menlo Park, CA, USA) was then advanced into the LA after proper flushing of the sheath. The choice of PFA catheter diameter size, 31 mm vs. 35 mm, was left to the operators depending on the size of the cardiac chamber and PV configurations.

The PFA catheter was then placed serially at the ostium of each PV in both the basket and flower configurations. At least two sets of PFA lesions were delivered for each position and configuration with circumferential rotation to deliver a nominal 8 PFA applications per PV. Additional PFA lesions may be delivered depending on 3D mapping to ensure acutely successful PVI and adequate antral coverage, as per the operators’ discretion. Each PFA application consists of 5 packets of pulses delivered over 2.5 s, which were not gated to the QRS complex. These lesions were delivered at 2.0 kV biphasic waveforms using the FARASTAR^TM^ PFA generator (Farapulse Boston Scientific Inc., Menlo Park, CA, USA). A post-ablation EAM may be performed.

Additional non-PV ablations may be performed using either PFA for LA PWA in select cases of pAF and persAF, as well as conventional radiofrequency (RF) ablation for patients with a history of (or induced) atrial flutter or frequent/incessant atrial tachycardia. Similarly, PWA was performed using 2.0 kV biphasic waveforms at 2 deliveries for each application site. The lesion set depended on the patient’s AF ablation history, LA size, and anatomy. Post PVI, 2 anchor lesions per vein extending to the LA posterior wall were deployed. A lesion set was then performed between the anchor lesions on the LA posterior wall (upper and lower row) with the catheter in a flower configuration and the intention of 50% overlap for the neighboring application sites on EAM with visualization of the PFA catheter. Fluoroscopy was used as an adjunct to corroborate the PFA catheter’s orientation and position, whenever necessary.

Electrical isolation was assessed using exit/entrance block testing with or without repeat LA EAM without a waiting period with the operator’s preferred catheter. In cases of residual or recovered PV or PW conductions, additional PFA lesions were delivered as required until PVI or PWA was confirmed on repeat assessment.

### 2.4. Endpoints

The primary efficacy endpoint was freedom from recurrence of any documented atrial tachyarrhythmias lasting at least 30 s after the 90-day blanking period. Atrial arrhythmia recurrence was detected using either a 24-h Holter, 3-day Multiday ECG, a preexisting cardiac implantable electronic device, or commercially approved handheld/ smart devices with ECG-capturing capabilities minimally at the 3-month and 1-year marks. Secondary endpoints include the need for repeat ablations, acute procedural success, and skin-to-skin procedural and fluoroscopy time. Safety endpoints included all-cause death within 30 days, cardiac tamponade, stroke, myocardial infarction, heart failure hospitalization, PNI, PV stenosis, and AEF.

### 2.5. Statistical Analysis

Statistical analysis was performed using SPSS version 29.0 (SPSS Inc., Chicago, IL, USA). Continuous variables were expressed as mean values with their associated standard deviations and were compared using Student’s *t*-test. Categorical variables were analyzed using Fisher’s exact test. Statistical significance was set at a p-value smaller than 0.05. A Kaplan–Meier analysis with a log-rank test was performed to compare the probability of freedom from atrial tachyarrhythmia between groups.

## 3. Results

### 3.1. Baseline Demographics and Procedural Characteristics

A total of 101 patients were enrolled between September 2022–January 2024. The patients were predominantly males (72.3%), with a mean age of 59.9 ± 9.1 years. Eighty (79.2%) patients had pAF, with a mean CHA2DS2-VASc score of 1.4 ± 1.3. Six patients had prior AF ablation using RF. Thirty-one (30.7%) patients had structural heart disease, of which ischemic heart disease (IHD) was the most common. Mean left ventricular ejection fraction (LVEF) was preserved at 57.4 ± 8.1%, and baseline use of AADs was 62.4%. Baseline characteristics for both the pAF and persAF groups are presented in Table 1.

Procedural characteristics are presented in Table 2. The 31 and 35 mm catheters were used in 86 and 15 procedures, respectively, with a higher likelihood of the larger sized catheter (35 mm) being used in the persAF group. Out of the 15 cases that utilized the 35 mm catheter, 11 were for dilated LA while 2 were for common ostium and the last 2 were for large PVs. The skin-to-skin procedural time was not significantly different between the pAF and persAF groups (116.8 ± 44.9 min vs. 137.0 ± 56.7 min, *p* = 0.164). Similarly, the fluoroscopy time and dose area product (DAP) were not significantly different between both groups (*p* = 0.550 and *p* = 0.372, respectively). The device LA dwell times for the pAF and persAF groups were 40.9 ± 14.3 min and 42.6 ± 13.0 min, respectively. EAM was used in 66 (82.5%) and 21 (100%) of cases in pAF and persAF, respectively. GA and ICE were used in 74 (73.3%) and 91 (90.1%) of the whole cohort, respectively. Almost half (42.6%) of the cohort required additional cavotricuspid isthmus (CTI) linear ablation using RF.

Total PFA applications required to achieve PVI were 44.2 ± 8.9 and 48.1 ± 12.4 for both pAF and persAF, respectively, with an average of 9–11 PFA applications per PV.

### 3.2. Additional PWA

Table 3 shows the pertinent baseline demographics and procedural characteristics, comparing patients in the PVI-only and those with additional PWA (PVI+PWA) groups. In our study, PFA for additional PWA was also performed in twenty-nine (28.7%) patients, of which eight (27.6%) were for PW scars, eight (27.6%) as first-line treatment based on patient history, i.e., persistent AF, four (13.8%) due to redo AF ablation, two (6.9%) for left atrium tachycardia or flutter, and seven (24.1%) for anatomical reasons, i.e., residual narrow gap of tissue on the posterior wall after PVI using PFA. Patients who underwent additional PWA were more likely to have lower mean LVEF, persistent AF, redo AF ablation as an indication, and GA and EAM usage, but less likely to have baseline AAD use (all *p* < 0.05).

In terms of procedural characteristics, there were no significant differences in device LA dwell time, skin-to-skin procedural duration, as well as fluoroscopy duration and dose between the PVI only and the PVI + PWA groups. In addition, the total number of PFA applications to achieve the acute PVI of all four pulmonary veins were similar in both groups.

Specific to the type of AF, the persAF group required a higher total number of PFA applications to achieve PWA compared to the pAF group (20.9 ± 5.8 vs. 15.1 ± 6.4, *p* = 0.015). See Table 2.

### 3.3. Outcomes and Follow-Up

Outcomes and follow-up are shown in Table 4. All cases had acutely successful PVI. There was one case of PVI failure from PFA due to technical issues with the FARASTARTM PFA generator (Farapulse-Boston Scientific Inc., Menlo Park, CA). PVI was completed using RF for this case.

**Figure 1:**
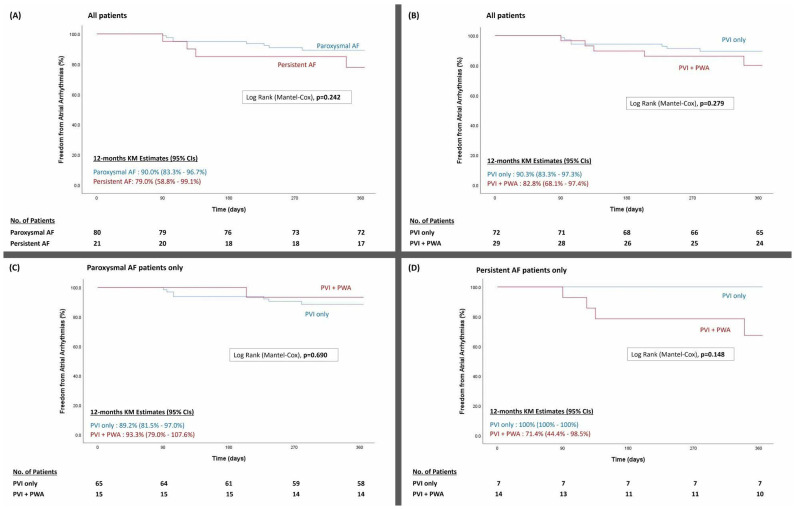
Freedom from any atrial arrhythmia recurrence after 90-day blanking period (**A**) paroxysmal AF versus persistent AF, (**B**) PVI only vs. PVI + PWA, (**C**) PVI only vs. PVI + PWA in paroxysmal AF cohort, and (**D**) PVI only vs. PVI + PWA in persistent AF cohort. AF denotes atrial fibrillation; KM, Kaplan–Meier; PVI, pulmonary vein isolation; PWA, posterior wall ablation.

Mean follow-up duration for the whole cohort was 415 ± 163 days. Recurrence rate of any atrial arrhythmia after 90-day blanking period was 10% and 21% for pAF and persAF, respectively, not achieving statistical significance with the available sample size (Figure 1A). Compared to PVI only, there was also no significant difference in primary efficacy endpoint in the PWI + PWA group (12-month KM estimates 90.3% [95% CI, 83.3–97.3] and 82.8% [95% CI, 68.1–97.4], respectively; Figure 1B). Within each of the pAF and persAF cohort, additional PWA did not affect freedom from atrial arrhythmia recurrence compared to PVI only (Figure 1C,D). There were no significant differences in all-cause mortality, heart failure hospitalization, and stroke between the pAF and persAF cohort as well as the PVI-only and PVI+PWA groups.

Of the twelve recurrences, three (25%) underwent redo ablation for AF (one case using RF and PFA each) and atrial flutter (one case using PFA). All three had reconnection at the right superior PV, while two had additional left superior PV reconnection. In the RF redone case, redo PVI was performed which terminated the AF. No further ablations were performed. For the other two cases, in addition to redo PVI, PWA, mitral isthmus, and CTI ablation using PFA was performed.

There were six (5.9%) inpatient complications, all of which were unrelated to the use of PFA. One patient developed cardiac tamponade during CTI RF ablation due to “steam-pop”, while one developed acute type 2 respiratory failure due to underlying OSA/OHS on chronic non-invasive ventilation, requiring monitoring and hospitalization. The remaining four cases were access-related, with groin/retroperitoneal hematoma requiring transfusion. There were two cases of all-cause mortality at 1 year, one of which occurred after 30 days and was unrelated to AF ablation. The other occurred during the same admission due to multiorgan failure from retroperitoneal hematoma/dissection from the manipulation of the ICE catheter. There were no reported occurrences of PV stenosis, PNI, or AEF in our cohort.

## 4. Discussion

In this study, our key findings are as follows:

- PFA can be implemented safely and effectively for PVI in both paroxysmal and persistent AF cases.
- Additional PWA to achieve successful PW isolation is feasible without any increase in procedural and fluoroscopy time or complications.
- The role and efficacy of durable PWA using PFA remains to be determined.
- A significant proportion of patients (42.6% of patients in our study) require additional CTI linear ablation. Larger studies on the safety of performing CTI ablation using PFA are required.

As shown in our study, all cases using PFA achieved acute PVI at the end of the procedure with an excellent 1-year freedom from atrial arrhythmia recurrence rate of 90.0% and 79.0% in the pAF and persAF groups, respectively. The recurrence rate was expectedly higher in the persAF group compared to the pAF group. The only randomized controlled trial (RCT) to date on PFA, the ADVENT trial, reported a 1-year freedom from primary composite endpoint in pAF patients of 73.3%, which was non-inferior to other thermal ablation sources [10]. This composite endpoint comprises initial procedural failure, documented atrial tachyarrhythmia after a 3-month blanking period, AAD use, cardioversion, or repeat ablation. Given that the definition for treatment failure was broader in the ADVENT trial, this explained the difference in 1-year outcome with our study. Other non-randomized retrospective and prospective studies reported a 1-year freedom from atrial arrhythmia recurrence rates of 66.2–90% for pAF and 60–79% for persAF [9,13,14,15], which were comparable to our study. However, differences in clinical study execution, primary endpoints, as well as nuances in various available PFA-based systems make it difficult to make direct comparisons.

Notably, our study provides procedural characteristics, supporting the similar efficiency of the PFA system in achieving PVI in the Asian region. Skin-to-skin procedural time, device LA dwell time, and fluoroscopic time for the whole cohort was 121.0 ± 48.0 min, 41.3 ± 14.0 min, and 28.7 ± 11.4 min, respectively. Compared to the ADVENT trial population, the skin-to-skin procedural and fluoroscopy time was shorter at 105.8 ± 29.4 min and 21.1 ± 11.0 min, but the LA dwell time was noticeably longer at 59.4 ± 18.3 min [10]. The ADVENT trial also demonstrated that procedural time and LA dwell time were significantly shorter with PFA compared to thermal sources, but at the expense of slightly longer fluoroscopic duration. The differences in procedural characteristics are likely explained by two factors. Firstly, the protocol-mandated 20 min waiting time in the ADVENT trial inflated the device LA dwell time in comparison to our study. Secondly, a significant proportion (42.6%) of our cohort required additional CTI ablation using RF, which increases procedural duration and the need for fluoroscopy. In the subset analysis of our study’s pAF patients undergoing PVI only without concomitant ablations (*n* = 49), the procedural characteristics nearly mirror that of the ADVENT trial, with a mean skin-to-skin procedural time, LA dwell time, and fluoroscopic time of 97.8 ± 36.5 min, 39.0 ± 13.0 min, and 25.6 ± 11.5 min, respectively. Similar trends in reduced procedural duration were also seen in other studies when comparing PFA to other thermal sources, but no differences in efficacy were noted when AF recurrences were considered [9,16,17,18].

Thus far, PFA has demonstrated a good safety profile with major complication rates ranging from 1 to 3.3% [7,8,13,19]. Our study reported an overall complication rate of 5.9%, of which, none of the complications were related to the use of PFA. Most pertinently, there was no evidence of PV stenosis, PNI, or AEF in our cohort. In the MANIFEST-PF registry involving the first 24 sites to utilize PFA in 1758 patients upon regulatory approval in Europe, the most common major complications observed were vascular injuries (0.3%) and pericardial tamponade (0.36%) [8]. One of the main factors for pericardial tamponade was attributed to the manipulation of the decapolar catheter from the coronary sinus to the right ventricle to provide backup pacing due to a significant vagal response during PFA delivery. During our pilot phase, 11 (84.6%) patients developed significant sinus pauses and/or hypotension requiring backup pacing. Thereafter, a vagolytic agent, atropine (600 micrograms), was commonly given 1–2 min prior to the initiation of PFA delivery in 77% of our cases to mitigate these vagal events. The expanded MANIFEST-17K study also showed that there was also an overall decreasing trend in the adverse events not just at the initial 24 sites of the MANIFEST-PF study, but also in other new sites enrolled in the study, highlighting that the safety of PFA use improves with greater operator experience as well as through “community-level global learning” due to mutual case sharing and proctorship [20].

While successful PVI remains the cornerstone of AF catheter ablation treatment, AF still recurs in up to 10–15% of pAF (and even higher in persAF) patients, despite durable PVI [21]. These recurrences are due to non-PV triggers for AF, one of which being the LA posterior wall. However, the role of additional posterior wall isolation (PWI) in persistent AF and redo AF ablation remains unclear at this juncture. RCTs evaluating PWI using thermal sources have yielded conflicting results, with larger studies such as POBI-AF and CAPLA demonstrating no benefit [22,23]. Similarly, a 2019 meta-analysis of three RCTs failed to show any incremental benefit of PWI, although the generalizability of the findings was limited by missing data, heterogenous patient population, differences in PWI approaches and definition, as well as applications of additional ablations beyond PVI and PWI [24]. Another key limitation of these studies lies in their inability to achieve 100% acute PWI, with successful PWI achieved in only 62–94% of the cases due to safety concerns on adjacent esophageal tissue damage limiting thermal energy delivery [22,23,25]. On the other hand, we achieved 100% acute PWA in all 29 cases with PFA, similar to findings from other early non-randomized studies using PFA for PWA [26,27,28].

Furthermore, the delivery of additional PWA in our study did not result in any significant lengthening in skin-to-skin procedural time, LA dwell time, and fluoroscopy time, despite a higher number of PFA applications compared to patients who underwent PVI only. We hypothesized that the similar procedural characteristics are contributed by several factors. Firstly, there was increased operator experience, as the first PWA was performed 6 months since inception of PFA at our center. Secondly, there was a lesser time needed to manipulate the pentaspline PFA catheter during flower mode for PVI in cases planned for additional PWA, as there was no concern in leaving an inadvertent narrow band of unablated posterior wall tissues, which may facilitate roof-dependent flutters. Additionally, the usage of EAM for all PWA cases and near routine ICE imaging at our center also possibly explains why additional PWA did not lengthen any of the procedural metrics. In one propensity-matched analysis of 556 patients undergoing ICE guided PFA for PVI only, Dello Russo et al. found no improvement in procedural metrics with ICE use [29]. However, the same group reported, in a separate cohort, which included patients who underwent additional PWA, that ICE-guided PFA showed improvements in skin-to-skin procedural time, fluoroscopy time, time to PVI, and total support time in patients who underwent additional PWA compared to those with PVI only [30].

Currently, there are no published RCTs evaluating the efficacy of PWA. Like thermal sources, current small non-randomized studies report 1-year atrial arrhythmia recurrence rates similar to that of thermal sources, being in the range of 17–37% [27,28]. Yet, a major difference between PFA and thermal sources lies in the durability of the PWA lesions. In one of these studies, durable PWA was seen in 85% of patients with atrial arrhythmia recurrence who underwent redo AF/AT ablation [27]. In a separate study, Reddy et al. reported an impressive 100% PWA durability during invasive remapping at 75 days [26]. These reported durability rates of PWA with PFA far overshadow the rates seen using thermal sources [22,23]. While the durability of PWA is crucial, whether this translates into a clinically significant reduction in atrial arrhythmia recurrence remains to be seen and warrants larger randomized studies to evaluate its impact.

There have been numerous studies demonstrating the positive impact of GA in reducing PV reconnection using thermal ablation due to better catheter stability [31,32]. Yet, the optimal sedation strategy, either deep sedation or GA in PFA, remains unclear. The use of GA for PFA remains widely varied, with the majority of US (56.1%) and European centers (80%) favoring deep sedation [9,20]. In contrast, deep sedation was only utilized in approximately one quarter of our cohort. This is likely explained by differences in institutional practices and availability of anesthesia support. Interestingly, the EUPORIA registry reported increasing GA usage with increased operator experience from 0% in operators with less than 2 years of experience to 23.1% in operators with more than 5 years of experience [9]. During our initial pilot phase of 13 patients, all but one patient underwent PFA under deep sedation using propofol and fentanyl. We noticed significant intraprocedural patient movement, as well as the frequent need for brief waiting periods from 30 s to 1 min between PFA deliveries due to transient hypotension. Since then, we have transitioned most of our PFA cases to be performed under GA. In a single center study, GA did not result in any significant differences in skin-to-skin procedural duration, total lab occupancy time, post-operative nausea and vomiting, and patient satisfaction compared to monitored anesthesia care with deep sedation [33]. However, the authors noted lower operative pain and chest movement, as well as greater operator satisfaction, with GA.

AF and atrial flutters commonly co-exist, with a reported concomitant atrial flutter prevalence of 20.6–25.9% in patients with AF [34,35]. Our study reports a much higher prevalence, with 42.6% of our patients requiring concomitant CTI ablation. Twenty-five (59.5%) had prior diagnosis of typical atrial flutter, while 12 (28.6%) had typical atrial flutter either induced post-PVI or organized from AF during PVI. The remaining five (11.9%) had empirical CTI ablation in view of underlying persAF/redo AF ablation. While there are emerging reports on the use of PFA for CTI ablation, there were some concerns about its safety, particularly from severe coronary spasms [36]. Hence, all CTI ablations were performed using RF in our center up until January 2024. The use of an additional RF catheter incurs additional costs for patients and may potentially be one of the major obstacles in hindering resource-conscious countries planning to adopt PFA technology. Fortunately, these coronary spasm adverse events can be mitigated using the administration of intravenous nitrates to facilitate successful PFA delivery to the CTI region [36], and will be one of our focus areas for future research.

### Limitations

This study has limitations which merit consideration. The nature of our prospective observational study design and small sample size limits direct causality and warrants interpretation in conjunction with other contemporaneous data by other groups. Moreover, the decision for additional PWA was not pre-specified and was left to operators’ discretion, limiting the generalizability of our results. Lastly, the follow-up of our study did not include continuous ECG monitoring, and thus transient episodes of atrial arrhythmia recurrence might not be captured.

## 5. Conclusions

Our study suggests that PFA can be safely, effectively, and efficiently adopted for AF ablation. Additional PWA to achieve acute successful PW isolation, if pursued, had similar procedural metrics to the PVI-only strategy without increases in procedural complications. The utility of PWA and CTI ablation using PFA warrants larger studies.

## Figures and Tables

**Table 1:** Baseline patient demographics.

Variables	Total (N = 101)	Paroxysmal AF (N = 80)	Persistent AF (N = 21)	*p*-Value
Age, y	59.9 ± 9.1	60.6 ± 9.3	57.4 ± 7.7	0.116
Male sex	73 (72.3)	55 (68.8)	18 (85.7)	0.172
Structural heart disease - IHD - HCM - TICMP - DCM - Others	31 (30.7) 14 (13.9) 6 (5.9) 5 (4.9) 3 (3.0) 3 (3.0)	23 (28.8) 13 (16.3) 5 (6.3) 3 (3.7) 1 (1.3) 1 (1.3)	8 (38.1) 1 (4.8) 1 (4.8) 2 (9.5) 2 (9.5) 2 (9.5)	1.000
Diabetes	11 (10.9)	9 (11.3)	2 (9.5)	1.000
Hypertension	34 (33.7)	27 (33.8)	7 (33.3)	1.000
Hyperlipidemia	44 (43.6)	36 (45.0)	8 (38.1)	0.628
Obstructive sleep apnea	9 (8.9)	5 (6.3)	4 (19.0)	0.087
CHA_2_DS_2_-VASc	1.4 ± 1.3	1.4 ± 1.4	1.1 ± 1.0	0.144
LVEF, %	57.4 ± 8.1	58.8 ± 6.4	52.2 ± 11.4	0.018
LA diameter, mm *	41.5 ± 7.3	40.7 ± 7.0	44.4 ± 7.8	0.077
LAVI, mL/m^2^ *	36.6 ± 15.1	34.5 ± 13.3	45.5 ± 18.8	0.029
Redo indication	6 (5.9)	3 (3.8)	3 (14.3)	0.102
Baseline AADs	63 (62.4)	56 (70.0)	7 (33.3)	0.004

Values are n (%) or mean ± SD. AADs denotes anti-arrhythmic drugs; AF, atrial fibrillation; DCM, dilated cardiomyopathy; HCM, hypertrophic cardiomyopathy; IHD, ischemic heart disease; LA, left atrium; LAVI, left atrial volume index; LVEF, left ventricular ejection fraction; TICMP, tachycardia induced cardiomyopathy. CHA2DS2-VASc scores range from 0 to 9, with higher scores indicating a greater risk of stroke. The highest score observed in this study was 5. * Data were available for 93 patients.

**Table 2:** Procedural characteristics.

Variables	Total (N = 101)	Paroxysmal AF (N = 80)	Persistent AF (N = 21)	*p*-Value
General anesthesia	74 (73.3)	55 (68.8)	19 (90.5)	0.054
ICE use	91 (90.1)	72 (90.0)	19 (90.5)	1.000
CTI ablation using RF	43 (42.6)	31 (38.8)	12 (57.1)	0.144
Additional ablations using RF - Mitral isthmus - LA substrate - Endocardial coronary sinus - Superior vena cava	11 (10.9) 3 (3.0) 2 (2.0) 1 (1.0) 5 (5.0)	8 (10.0) 2 (2.5) 2 (2.5) 1 (1.3) 3 (3.8)	3 (14.3) 1 (4.8) 0 (0.0) 0 (0.0) 2 (9.5)	0.469
EAM use - CARTO - Ensite - Rhythmia	87 (86.1) 19 (18.8) 63 (62.4) 5 (4.9)	66 (82.5) 12 (15.0) 50 (62.5) 4 (5.0)	21 (100.0) 7 (33.3) 13 (61.9) 1 (4.8)	0.038
Device Size - 31 mm - 35 mm	86 (85.1) 15 (14.9)	73 (91.3) 7 (8.8)	13 (61.9) 8 (38.1)	0.003
Device left atrial dwell time, min	41.3 ± 14.0	40.9 ± 14.3	42.6 ± 13.0	0.602
Skin-to-skin procedural time, min	121.0 ± 48.0	116.8 ± 44.9	137.0 ± 56.7	0.164
Fluoroscopy time, min	28.7 ± 11.4	28.3 ± 11.5	30.0 ± 11.1	0.550
Fluoroscopy DAP, Gy.cm^2^	13,591 ± 10,414	13,050 ± 10,240	15,612 ± 11,093	0.372
Fluoroscopy skin dose AK, Gy	133 ± 97	127 ± 91	158 ± 114	0.281
Vagal response	15 (14.9)	14 (17.5)	1 (4.8)	0.185
Atropine use	77 (76.2)	60 (75.0)	17 (81.0)	0.775
PWA	29 (28.7)	15 (18.8)	14 (66.7)	<0.001
Total PFA applications	50.1 ± 13.8	47.1 ± 11.2	61.3 ± 16.7	0.001
Total PFA applications for PVI	45.0 ± 9.8	44.2 ± 8.9	48.1 ± 12.4	0.190
- LSPV	10.0 ± 3.2	10.1 ± 3.2	9.5 ± 3.6	0.497
- LIPV	9.3 ± 2.9	9.5 ± 2.8	8.4 ± 3.1	0.144
- RSPV	10.3 ± 3.0	10.1 ± 3.0	11.1 ± 3.4	0.257
- RIPV	10.5 ± 3.3	10.7 ± 3.4	9.5 ± 3.2	0.147
- PW	17.9 ± 6.9	15.1 ± 6.4	20.9 ± 5.8	0.015

Values are n (%) or mean ± SD. AF denotes atrial fibrillation; AK, air kerma; CTI, cavotricuspid isthmus; DAP, dose area product; EAM, electroanatomic map; ICE, intracardiac echocardiography; LA, left atrium; LIPV, left inferior PV; LSPV, left superior PV; PFA, pulse field ablation; PVI, pulmonary vein isolation; PW, posterior wall; PWA, posterior wall ablation; RF, radiofrequency; RIPV, right inferior PV; RSPV, right superior PV.

**Table 3:** Pertinent baseline demographics and procedural characteristics between the PVI-only and PVI + PWA cohort.

Variables	Total (N = 101)	PVI Only (N = 72)	PVI + PWA (N = 29)	*p*-Value
LVEF, %	57.4 ± 8.1	58.9 ± 6.1	53.7 ± 10.9	0.020
LA diameter, mm ^#^	41.5 ± 7.3	40.5 ± 7.1	43.6 ± 7.5	0.071
LAVI, mL/m^2 #^	36.6 ± 15.1	34.8 ± 13.1	40.9 ± 18.4	0.121
Baseline AADs	63 (62.4)	50 (69.4)	13 (44.8)	0.025
Persistent AF	21 (20.8)	7 (9.7)	14 (48.3)	<0.001
Redo indication	6 (5.9)	1 (1.4)	5 (17.2)	0.007
General anesthesia	74 (73.3)	47 (65.3)	27 (93.1)	0.005
ICE use	91 (90.1)	64 (88.9)	27 (93.1)	0.720
CTI ablation using RF	43 (42.6)	28 (38.9)	15 (51.7)	0.271
Additional ablations using RF - Mitral isthmus line - LA substrate - Endocardial coronary sinus - Superior vena cava	11 (10.9) 3 (3.0) 2 (2.0) 1 (1.0) 5 (5.0)	5 (6.9) 2 (2.8) 1 (1.4) 0 (0.0) 2 (2.8)	6 (20.7) 1 (3.4) 1 (3.4) 1 (3.4) 3 (10.3)	0.192
EAM use - CARTO - Ensite - Rhythmia	87 (86.1) 19 (18.8) 63 (62.4) 5 (5.0)	58 (80.6) 11 (15.3) 42 (58.3) 5 (6.9)	29 (100.0) 8 (27.6) 21 (72.4) 0 (0.0)	0.009
PFA Size - 31 mm - 35 mm	86 (85.1) 15 (14.9)	64 (88.9) 8 (11.1)	22 (75.9) 7 (24.1)	0.123
Device LA dwell time, min	41.3 ± 14.0	40.7 ± 14.6	42.7 ± 12.6	0.482
Skin-to-skin procedural time, min	121.0 ± 48.0	117.4 ± 48.7	130.0 ± 47.4	0.259
Fluoroscopy time, min	28.7 ± 11.4	28.8 ± 12.8	28.2 ± 6.9	0.777
Fluoroscopy DAP, Gy.cm^2^	13,591 ± 10,414	13,466 ± 10,297	13,916 ± 10,925	0.860
Total skin dose AK, Gy	133 ± 97	136 ± 98	126 ± 96	0.648
Total PFA applications	50.1 ± 13.8	43.9 ± 9.5	65.5 ± 10.2	<0.001
Total PFA applications for PVI	45.0 ± 9.8	43.9 ± 9.5	47.7 ± 10.2	0.084
- LSPV	10.0 ± 3.2	10.0 ± 3.2	9.9 ± 3.5	0.912
- LIPV	9.3 ± 2.9	9.4 ± 2.7	9.0 ± 3.3	0.532
- RSPV	10.3 ± 3.0	10.0 ± 2.9	11.2 ± 3.2	0.084
- RIPV	10.5 ± 3.3	10.4 ± 3.4	10.5 ± 3.2	0.942
- PW	17.9 ± 6.7	N/A	17.9 ± 6.7	N/A

Values are n (%) or mean ± SD. AF denotes atrial fibrillation; AK, air kerma; CTI, cavotricuspid isthmus; DAP, dose area product; EAM, electroanatomic map; ICE, intracardiac echocardiography; LA, left atrium; LIPV, left inferior PV; LSPV, left superior PV; PFA, pulse field ablation; PVI, pulmonary vein isolation; PW, posterior wall; PWA, posterior wall ablation; RF, radiofrequency; RIPV, right inferior PV; RSPV, right superior PV. ^#^ Data were available for 93 patients.

**Table 4:** Outcomes and follow-up in paroxysmal and persistent AF.

Variables	Total (N = 101)	Paroxysmal AF (N = 80)	Persistent AF (N = 21)	*p*-Value	PVI only (N = 72)	PVI + PWA (N = 29)	*p*-Value
Acutely successful PVI	101 (100.0)	80 (100.0)	21 (100.0)	1.000	72 (100.0)	29 (100.0)	1.000
Inpatient complications - Cardiac tamponade - Acute respiratory failure - Access bleeding/hematoma - Retroperitoneal hematoma	6 (5.9) 1 (1.0) 1 (1.0) 3 (3.0) 1 (1.0)	5 (6.3) 1 (1.3) 1 (1.3) 2 (2.5) 1 (1.3)	1 (4.8) 0 (0.0) 0 (0.0) 1 (4.8) 0 (0.0)	0.635	5 (6.9) 1 (1.4) 1 (1.4) 2 (2.8) 1 (1.4)	1 (3.4) 0 (0.0) 0 (0.0) 1 (3.4) 0 (0.0)	0.670
Follow-up duration, days	415 ± 163	425 ± 169	376 ± 132	0.161	439 ± 177	355 ± 97	0.003
All-cause mortality	2 (2.0)	2 (2.5)	0 (0.0)	1.000	2 (2.8)	0 (0.0)	1.000
Stroke	0 (0.0)	0 (0.0)	0 (0.0)	N/A	0 (0.0)	0 (0.0)	N/A
Myocardial infarction	0 (0.0)	0 (0.0)	0 (0.0)	N/A	0 (0.0)	0 (0.0)	N/A
Heart failure hospitalization	2 (2.0)	1 (1.3)	1 (4.8)	0.374	1 (1.4)	1 (3.4)	0.494
12-month arrhythmia recurrence ǂ	12 (11.9)	8 (10.0)	4 (19.0)	0.257	7 (9.7)	5 (17.2)	0.506
AAD at follow-up ǂ	15 (14.9)	13 (16.3)	2 (9.5)	0.728	11 (15.3)	4 (13.8)	0.770
Redo ablation ǂ	3 (3.0)	3 (3.8)	0 (0.0)	0.500	3 (4.2)	0 (0.0)	0.549

Values are n (%) or mean ± SD. AADs denote anti-arrhythmic drugs; AF, atrial fibrillation; PVI, pulmonary vein isolation; PWA, posterior wall ablation. Outcomes were measured at the 12 month mark. ǂ Data were available for 93 patients.

## Data Availability

Data is contained within the article.
